# Initial Experience With Transcatheter Aortic Valve Replacement in Patients With Severe Aortic Stenosis in Nicaragua

**DOI:** 10.7759/cureus.106149

**Published:** 2026-03-30

**Authors:** Ivonne Leytón, Jairo Campos, Erick Gutierrez, Itza López, Juan Cristobal Mendoza

**Affiliations:** 1 Public Health Sciences, Universidad Americana, Managua, NIC; 2 Medicine and Surgery, Universidad Americana, Managua, NIC; 3 Medicine, Universidad Americana, Managua, NIC; 4 General Medicine, Universidad Americana, Managua, NIC; 5 Clinical Department, Hospital Militar Escuela Dr. Alejandro Davila Bolaños, Managua, NIC

**Keywords:** aortic stenosis, aortic valve, surgical replacement of valve, tavr, valve efficacy, varc-3

## Abstract

Background

Aortic stenosis is a progressive valvular disease strongly associated with aging and high mortality once symptoms develop. Surgical aortic valve replacement has traditionally been the standard treatment for symptomatic severe disease; however, transcatheter aortic valve replacement (TAVR) has emerged as an effective, less invasive alternative for patients with increased surgical risk, supported by evidence from major clinical trials. Since its introduction in Nicaragua, procedural volume has steadily increased. This study aimed to describe the initial national experience with TAVR in patients with severe aortic stenosis in Nicaragua.

Materials and methods

This retrospective descriptive case series included 16 patients with confirmed severe aortic stenosis who underwent TAVR between July 2023 and March 2024 at a single center in Managua, Nicaragua. Baseline clinical and echocardiographic data were obtained from medical records. Outcomes were evaluated using Valve Academic Research Consortium-3 (VARC-3) criteria, including technical success, device success, and early safety at 30 days. Data were analyzed using IBM SPSS Statistics 25 (IBM Corp., Armonk, NY) with categorical variables reported as n (%), continuous variables as mean ± SD, and pre- versus post-procedural mean transaortic pressure gradients compared using the Wilcoxon signed-rank test. Statistical significance was defined as p ≤ 0.05, which was calculated through Fisher's exact test (dichotomous variables) and Fisher-Freeman-Halton exact test (polytomous variables). Institutional review board approval was obtained.

Results

The majority of participants were in the 61-70 year age group, accounting for 7 (43.8%) patients, with a mean age of 68.9 ± 8.7 years; 9 (56.3%) were male, and 9 (56.3%) were overweight. The most prevalent comorbidities were hypertension in 15 (93.8%) and diabetes mellitus in 6 (37.5%). Dyspnea was the most common symptom, reported in 15 (93.8%), followed by heart failure in 9 (56.3%) and angina in 8 (50%). Baseline hemodynamics showed a mean transaortic gradient of 55.7 ± 21.1 mmHg, with 14 (87.5%) presenting gradients >40 mmHg, and a mean aortic valve area of 0.68 ± 0.3 cm². Self-expanding valves were implanted in 15 (93.8%), predominantly Acurate Neo 2 in 13 (81.3%) (Boston Scientific Corp., Marlborough, MA). Overall, 13 (81.3%) procedures met VARC-3 criteria for technical success. No intraprocedural or 30-day deaths were recorded. At 30 days, device success was achieved in 12 (75%), early safety in 15 (93.8%), and intended valve performance was satisfactory in 14 (87.5%), with a post-procedural mean gradient of 9.08 ± 6.3 mmHg and no moderate-to-severe aortic regurgitation. The median transaortic pressure gradient decreased from baseline to 30 days after TAVR, from 52.5 (IQR 41.5-67.5) to 9.0 (IQR 3.47-11), with p<0.001 indicating a statistically significant change and a substantial clinical impact on patients’ hemodynamic status. Permanent pacemaker implantation and paravalvular leak occurred in 1 (6.3%) patient each.

Conclusion

In this initial single-center experience in Nicaragua, TAVR was feasible and achieved favorable short-term outcomes, with significant improvement in transaortic hemodynamics at 30 days. These findings support the early safety and effectiveness of TAVR in this setting, although larger prospective studies are needed to confirm results and improve generalizability.

## Introduction

Aortic stenosis is defined as a narrowing of the aortic valve that obstructs blood outflow from the left ventricle to the aorta, detected through clinical manifestations and echocardiographic findings, including mean transaortic pressure gradient >40 mmHg, peak aortic velocity >4 m/s, and AVA < 1.0 cm^2^ [1]. The development of this disease is strongly associated with aging, affecting approximately 4.6% of individuals older than 75 years, with prevalence increasing further in more advanced age groups [2]. On the other hand, the most common underlying cause in younger patients is a bicuspid aortic valve, in which chronic mechanical stress accelerates valvular degeneration [3].

Beyond its epidemiological significance, understanding the pathophysiology of the disease is essential to elucidate its clinical progression. Initially, sclerosis of the valve occurs, followed by stenosis, and may occur without hemodynamically relevant obstruction; however, approximately 10-15% of patients progress to clinically meaningful disease over time [2]. Clinical practice guidelines describe the classic symptomatology as dyspnea, angina, syncope, heart failure, and a systolic murmur best heard at the aortic area, with diagnosis confirmed through echocardiographic evaluation [1]. These clinical and echocardiographic findings form the basis for disease staging. Accordingly, aortic stenosis is classified into four stages, ranging from patients at risk (Stage A) to symptomatic severe disease (Stage D), in which annual mortality may reach approximately 25% if left untreated [2].

Given the progressive nature and associated mortality of advanced disease, risk stratification is appropriate to guide therapeutic decision-making, ranging from conservative measures to definitive aortic valve replacement [1]. Although surgical aortic valve replacement (SAVR) has traditionally been the first-line treatment for symptomatic severe aortic stenosis, a substantial proportion of patients are not suitable candidates due to procedural risk, which can be assessed using validated tools such as the Society of Thoracic Surgeons (STS) score and EuroSCORE II [1]. In this context, transcatheter aortic valve replacement (TAVR) has emerged as the primary alternative for patients with high surgical risk and has progressively gained widespread adoption due to improvements in safety and efficacy demonstrated in major clinical trials. Initial evidence from the PARTNER 1B trial (2010) demonstrated a clear clinical benefit of TAVR compared with standard medical therapy in inoperable patients with severe aortic stenosis [4]. Subsequently, the PARTNER 1A showed noninferiority in one-year mortality compared with SAVR in high-risk surgical candidates [5]. This indication was later extended to intermediate-risk patients following the PARTNER 2A trial, which demonstrated noninferiority for the composite endpoint of death and disabling stroke at two years [6]. More recently, the PARTNER 3 trial established the efficacy of TAVR in low-risk patients, showing superiority at one year and sustained comparable outcomes over longer-term follow-up [7-9].

Despite the strong body of international evidence supporting TAVR, its implementation in low- and middle-income countries remains limited. In Nicaragua, TAVR was introduced recently, and since its introduction, the number of procedures has progressively increased [10]. The aim of this study was to describe the initial national experience with TAVR in patients with severe aortic stenosis with high surgical risk in Nicaragua.

## Materials and methods

Study design

This is a single-center, descriptive study of retrospectively collected data, wherein 16 patients with a diagnosis of severe aortic stenosis who underwent TAVR were included, in the period from July 2023 to March 2024. This study was conducted at the Hospital Militar Escuela "Alejandro Dávila Bolaños", in Managua, Nicaragua. Their preoperative, intraoperative, and postoperative outcomes were collected. Approval for the study was requested and granted by the institutional review board, strictly preserving all the bioethical principles.

Selection criteria

The inclusion criteria were patients with a confirmed diagnosis of severe aortic stenosis (based on clinical manifestations and echocardiographic findings such as the mean transaortic pressure gradient and aortic valve area) with high surgical risk, who underwent TAVR between July 2023 and March 2024, and patients with a documented 30-day follow-up after the procedure. The exclusion criteria were patients without a confirmed diagnosis of severe aortic stenosis, patients who did not undergo TAVR, patients treated by an interventional cardiologist outside Hospital Militar Escuela “Alejandro Dávila Bolaños,” and patients who did not have a 30-day follow-up by the hospital's cardiology team.

Definitions

Clinical and echocardiographic data were obtained from the clinical record. The baseline clinical variables that were included were age, gender, body mass index (BMI), comorbidities, dyspnea, angina, syncope, and heart failure. Additionally, the echocardiographic variables that were included were the mean transaortic pressure gradient before and after the procedure, and the aortic valve area. The outcomes of the TAVR were evaluated through technical success (immediate), device success (at 30 days), and early safety (at 30 days) criteria, proposed by the Valve Academic Research Consortium 3 (VARC-3).

Data analysis

Data were processed in IBM SPSS Statistics 25 (IBM Corp., Armonk, NY). Categorical variables were expressed in frequencies and percentages, and quantitative variables were summarized in means and standard deviations. Categorical variables were analyzed using the Fisher exact test for dichotomous variables, and Fisher-Freeman-Halton test for polytomous variables, due to a small sample size and small cell counts. Normal distribution of the data was assessed through the Shapiro-Wilk test. However, considering the limited sample size and to ensure robustness of the analysis, paired comparison of the mean transaortic pressure gradient before and after the procedure was conducted using the Wilcoxon signed-rank test. A p-value ≤ 0.05 was considered statistically significant.

## Results

Preoperative characteristics

The age distribution of the patients was mainly between 61 and 70 years, accounting for 7 (43.8%) individuals, with a mean age of 68.9 ± 8.7 years. Male patients comprised 9 (56.3%) of the study population. Regarding nutritional status, overweight was the most frequent category, observed in 9 (56.3%) patients. The most prevalent comorbidities were hypertension in 15 (93.8%), diabetes mellitus in 6 (37.5%), and chronic kidney disease in 3 (18.8%) patients (Table 1).

**Table 1:** Demographics and Baseline Characteristics Results are presented as absolute and relative frequencies, n (%). Nutritional status data were not available for one patient.

Variable	Frequency (n=16)	Percentage
Age Groups		
50 to 60 years	3	18.8
61 to 70 years	7	43.8
71 to 80 years	4	25
Over 80 years	2	12.5
Sex		
Female	7	43.8
Male	9	56.3
Comorbidities		
Hypertension	15	93.8
Diabetes type 2	6	37.5
Degenerative osteoarthritis	1	6.3
Spondyloarthrosis of the spine	1	6.3
Coronary artery disease	2	12.5
Mitral regurgitation	3	18.8
Mitral stenosis	1	6.3
Chronic kidney disease	3	18.8
Hepatic steatosis	1	6.3
Chronic alcoholism	1	6.3
Smoking history	2	12.5
Hypothyroidism	2	12.5
Rheumatoid arthritis	1	6.3
Nutritional Status		
Eutrophic	3	20
Obesity	3	20
Overweight	9	60

Clinically, the most common presenting symptom was dyspnea, reported in 15 (93.8%) patients, followed by angina in 8 (50%), syncope in 4 (25%), and heart failure in 9 (56.3%). The mean transaortic pressure gradient was 55.7 ± 21.1 mmHg, with 14 (87.5%) patients presenting gradients above 40 mmHg. The mean aortic valve area was 0.68 ± 0.3 cm² (Table 2).

**Table 2:** Clinical Presentation Results are presented as absolute and relative frequencies, n (%).

Variable	Frequency (n=16)	Percentage
Dyspnea	15	93.8
Angina	8	50
Syncope	4	25
Heart failure	9	56.3
Mean transaortic pressure gradient before procedure		
Over 40 mmHg	14	87.5
Less than 40 mmHg	2	12.5

Intraoperative characteristics

Self-expanding valves were the most frequently implanted devices, used in 15 (93.8%) patients. The predominant valve was the Acurate Neo 2 (Boston Scientific Corp., Marlborough, MA), implanted in 13 (81.3%) cases, followed by the Evolut PRO 2 in 1 (6.3%) and the Evolut PRO+ in 1 (6.3%) (Medtronic Inc., Minneapolis, MN). A balloon-expandable valve was used in 1 (6.3%) patient, corresponding to the Myval device (Meril Life Sciences, Vapi, India). The mean prosthetic valve size was 25.4 ± 1.78 mm. No intraprocedural deaths were recorded. Successful vascular access and correct valve positioning were achieved in 15 (93.8%) patients. Additional intraprocedural interventions were required in 3 (18.8%) cases, consisting of one valve-in-valve implantation due to prosthesis displacement, and two vascular repair procedures in the femoral arteries. Overall, 13 (81.3%) procedures met the VARC-3 criteria for technical success (Table 3).

**Table 3:** Intraoperative characteristics Results are presented as absolute and relative frequencies, n (%). Technical success as defined by the Valve Academic Research Consortium-3 (VARC-3) consensus criteria.

Variables	Frequency (n=16)	Percentage
Type of Valve		
Auto-expandable	15	93.8
Balloon-expandable	1	6.3
Valve Model		
Acurate Neo 2	13	81.3
Evolut PRO	1	6.3
Evolut PRO Plus	1	6.3
Myval	1	6.3
Technical success	13	81.3
Mortality	0	0
Successful access	15	93.8
Correct valve positioning	15	93.8
Surgery or intervention related to the device	3	18.8

Postoperative characteristics

At 30 days post-procedure, VARC-3 recommends assessing two key domains: device success and early safety. Device success was achieved in 12 (75%) patients. No deaths occurred during follow-up. Valve performance was satisfactory in 14 (87.5%) patients, with a post-procedural mean transaortic pressure gradient of 9.08 ± 6.3 mmHg; 14 (87.5%) patients had residual gradients below 20 mmHg, and none presented moderate-to-severe aortic regurgitation (Table 4). The median transaortic pressure gradient decreased from baseline to 30 days after TAVR from 52.5 (IQR 41.5-67.5) to 9.00 (IQR 3.47-11) with p<0.001 showing statistical significance per the Wilcoxon signed-rank test, showing a great clinical impact on the hemodynamic status of the patient. Regarding early safety, 15 (93.8%) patients met VARC-3 criteria at 30 days. No cerebrovascular events were recorded, and only 1 patient experienced stage 3-4 acute kidney injury or VARC-3 type 2-4 bleeding. Only 1 (6.3%) patient required permanent pacemaker implantation, and paravalvular leak was observed in one case (6.3%) (Table 4).

**Table 4:** Outcomes 30 Days After Procedure Results are presented as absolute and relative frequencies, n (%). Device success and early safety as defined by the Valve Academic Research Consortium-3 (VARC-3) consensus criteria.

Variables	Frequency (n=16)	Percentage
Device success	12	75
Mortality 30 days	0	0
Surgery or intervention related to the device	1	6.3
Intended performance of the valve	14	87.5
Mean transaortic pressure gradient at 30 days		
≥ 20 mmHg	2	12.5
< 20 mmHg	14	87.5
Less than moderate aortic regurgitation	16	100
Early safety	15	93.8
Stroke	0	0
VARC type 2-4 bleeding	0	0
Type of bleeding	N/A	N/A
Major vascular complication	0	0
Acute kidney disease stage 3 or 4	1	6.3
Moderate or severe aortic regurgitation	0	0
New permanent pacemaker	1	6.3
Paravalvular leakage	1	6.3

The highest intended valve performance rate was observed among patients older than 70 years, with 6 (100%) meeting this endpoint. Likewise, valve efficacy was achieved in 1 (100%) patient who received a balloon-expandable valve, specifically the Myval valve. However, these differences were not statistically significant (Table 5). Similarly, the highest device success rate was recorded in patients older than 70 years, corresponding to 6 (100%), and in the patient treated with the Myval balloon-expandable valve, with 1 (100%), also without statistical significance (Table 6). In the same subgroup, early safety at 30 days was achieved in 6 (100%) patients older than 70 years, as well as the age group of 50 to 60 years, with 3 (100%) patients, without statistically significant differences (Table 7).

**Table 5:** Intended Valve Performance by Age Group, Type of Valve, and Valve Model Bivariate analysis of intended valve performance as defined by the Valve Academic Research Consortium-3 (VARC-3) consensus criteria, according to age group and valve-related characteristics. Results are presented as absolute and relative frequencies, n (%). P-values were calculated using Fisher's exact test for dichotomous variables and Fisher-Freeman-Halton exact test for polytomous variables, with p≤0.05 to be considered statistically significant.

Variable	Intended Valve Performance, N=14, n(%)			p-value
					Age Groups				
50 to 60 years	2 (66.7%)			0.767
61 to 70 years	6 (85.7%)			
71 to 80 years	4 (100%)			
Above 80 years	2 (100%)			
Type of Valve				
Auto-expandable	13 (86.7%)			1
Balloon-expandable	1 (100%)			
Valve Model				
Acurate Neo 2	11 (84.6%)			1
Evolut PRO	1 (100%)			
Evolut PRO Plus	1 (100%)			
Myval	1 (100%)			

**Table 6:** Device Success by Age Groups, Type of Valve and Valve Model Bivariate analysis of device success as defined by the Valve Academic Research Consortium-3 (VARC-3), according to age group and valve-related characteristics. Results are presented as absolute and relative frequencies, n (%).  P-values were calculated using Fisher's exact test for dichotomous variables and Fisher-Freeman-Halton exact test for polytomous variables with p≤0.05 to be considered statistically significant.

Variables	Device Success, N=12, n(%)			p-value
					Age Groups				
50 to 60 years	2 (66.7%)			0.392
61 to 70 years	4 (57.1%)			
71 to 80 years	4 (100%)			
Above 80 years	2 (100%)			
Type of Valves				
Auto-expandable	11 (73.3%)			1
Balloon-expandable	1 (100%)			
Valve Model				
Acurate Neo 2	9 (69.2%)			1
Evolut PRO	1 (100%)			
Evolut PRO Plus	1 (100%)			
Myval	1 (100%)			

**Table 7:** Early Safety by Age Groups Bivariate analysis of early safety as defined by the Valve Academic Research Consortium-3 (VARC-3), according to age group and valve-related characteristics. Results are presented as absolute and relative frequencies, n (%). P-values were calculated using Fisher's exact test for dichotomous variables and Fisher-Freeman-Halton exact test for polytomous variables, with p≤0.05 to be considered statistically significant.

Age Groups	Early Safety, N=15, n(%)			p-value
					50 to 60 years	3 (100%)			1
61 to 70 years	6 (85.7%)								
71 to 80 years	4 (100%)								
Above 80 years	2 (100%)								

## Discussion

The average age of the study population was 68 years, with the predominant age group being 61 to 70 years. This result differs from the findings reported in international studies conducted in Italy and other European countries that studied populations with an average age of over 80 years. The difference may be explained by demographic variations, including lower life expectancy in Nicaragua compared to those populations [11-13]. Despite these variations, aging remains one of the most important risk factors for developing this disease, and that is why this population is the main target for this type of procedure [2].

The majority of the population was male; however, prior studies suggest no significant sex based differences in the incidence or mortality of aortic stenosis, particularly in populations over 50 years of age, where the epidemiological risks associated with these conditions tend to be equal between men and women [14].

The most prevalent comorbidities were systemic arterial hypertension and diabetes mellitus, both of which have pathophysiological mechanisms that directly influence the onset and progression of aortic stenosis and other cardiovascular diseases. This is particularly relevant when these conditions are associated with other clinical factors, such as nutritional status, where the majority of the patients in this study were overweight and obese. From an etiopathogenic perspective, the overlap of risk factors between aortic stenosis and atherosclerosis means that the pathophysiological processes of both conditions are practically identical [2]. When analyzing the clinical characteristics of the patients, it was found that the most commonly reported symptom was dyspnea, followed by heart failure, angina, and syncope, consistent with the classic clinical presentation of this heart valve disease [2].

The TAVR procedure involves aortic valve replacement using less invasive techniques than open-heart surgery, primarily for patients with severe aortic stenosis and high surgical risk. In this study, the vast majority of patients had a mean transaortic pressure gradient greater than 40 mmHg, with an average of 55.7 mmHg, and the mean aortic valve area was 0.68 cm², both parameters indicating severe disease, consistent with the criteria for intervention. Traditionally, another determining factor when deciding whether to do the intervention or not is whether the patient is symptomatic. Recent data from a systematic review and meta-analysis based on four randomized controlled trials, which compared early intervention vs clinical surveillance in asymptomatic patients with severe aortic stenosis, support that there was a significant reduction in unplanned cardiovascular or heart failure hospitalization in the early intervention group, without significant differences in all-cause and cardiovascular mortality [15].

The most commonly used valve type in our series was the self-expanding platform, particularly the Acurate Neo 2. The choice between balloon-expandable and self-expanding valves is influenced by several factors, including institutional availability, patient anatomy, procedural planning, and operator experience. However, contemporary comparative evidence suggests that balloon-expandable valves may be associated with lower perioperative and 30-day mortality, lower rehospitalization rates, and a reduced need for permanent pacemaker implantation, while no significant differences have been consistently observed in stroke, bleeding, acute kidney injury, or myocardial infarction, as demonstrated in a recent systematic review and meta-analysis [16].

The primary outcomes in our study were defined according to the VARC-3 criteria, including intraoperative technical success and 30-day post-procedural outcomes (device success and early safety) [17]. Overall, the majority of patients achieved favorable results across these endpoints, particularly within the early safety domain. These findings are consistent with international reports describing early experiences using similar self-expanding valve platforms [11-12].

From a hemodynamic standpoint, procedural performance was also satisfactory. The median transaortic pressure gradient decreased from baseline to 30 days after TAVR from 52.5 (IQR 41.5-67.5) to 9.00 (IQR 3.47-11) with p<0.001 showing statistical significance per the Wilcoxon signed-rank test, reflecting substantial hemodynamic improvement. These results align with randomized trial meta-analytic evidence showing that TAVR is associated with favorable hemodynamic performance, including larger effective orifice areas, lower residual gradients, and lower rates of prosthesis-patient mismatch compared with surgical valve replacement, although at the expense of higher rates of paravalvular regurgitation [18].

Limitations of the study should be acknowledged. TAVR remains a relatively new procedure in Nicaragua, with less than three years of national experience. Consequently, this study represents a single-center descriptive case series with a small sample size, which limits statistical power and the generalizability of the findings. In addition, clinical documentation and procedural reporting were not fully standardized during the early implementation phase, resulting in variability in the completeness of baseline and follow-up data across patients. Despite these limitations, our findings provide an initial national perspective on the feasibility, safety, and hemodynamic performance of TAVR in Nicaragua during the early adoption period.

## Conclusions

In this initial single-center experience with TAVR in Nicaragua, the procedure was successfully implemented in a relatively younger population compared with most European cohorts, with a high prevalence of cardiovascular risk factors and predominantly symptomatic severe aortic stenosis. Overall, early outcomes assessed using VARC-3 definitions were favorable, and the intervention resulted in a significant and clinically meaningful improvement in transaortic hemodynamics at 30 days. Despite the limitations in the early implementation phase of this novel program, these findings support the feasibility, short-term safety, and hemodynamic effectiveness of TAVR in Nicaragua, while highlighting the need for standardized clinical reporting and larger prospective studies to confirm outcomes and expand generalizability.
